# Functional Characterization of Dark Sleeper (*Odontobutis obscura*) TBK1 on IFN Regulation

**DOI:** 10.3389/fimmu.2019.00985

**Published:** 2019-05-03

**Authors:** Jian Chen, Zhuo Cong Li, Long Feng Lu, Pei Li, Xi-Yin Li, Shun Li

**Affiliations:** ^1^Fisheries Research Institute, Wuhan Academy of Agricultural Sciences, Wuhan, China; ^2^State Key Laboratory of Freshwater Ecology and Biotechnology, Institute of Hydrobiology, Chinese Academy of Sciences, Wuhan, China; ^3^University of Chinese Academy of Sciences, Beijing, China; ^4^Key Laboratory of Aquaculture Disease Control, Ministry of Agriculture, Wuhan, China

**Keywords:** TBK1, IFN, RLRs, antiviral, *Odontobutis obscura*

## Abstract

In East Asia, the dark sleeper, *Odontobutis obscura* (*O. obscura*) is a crucial commercial species of freshwater fish; however, its molecular biology research is still undeveloped, including its innate immune system, which is pivotal to antiviral responses. In this study, we cloned and identified the characterization and kinase function of dark sleeper TANK-binding kinase 1 (TBK1), supplementing the evidence of the conservation of this classical factor in fish. First, the ORF of *Odontobutis obscurus* (*O. obscura*) TBK1 (OdTBK1) was cloned from liver tissue by RACE-PCR. Subsequent nucleic acid and amino acid sequence analysis suggested that OdTBK1 is homologous with other fish TBK1, and the N-terminal Serine/Threonine protein kinases catalytic domain (S_TKc) and C-terminal coiled coil domain (CCD) are conserved. Subsequently, the cellular distribution demonstrated that OdTBK1 was located in the cytoplasm region. With regard to the identification of functions, OdTBK1 activated several interferon (IFN) promoters' activity and induced downstream IFN-stimulated genes (ISGs) expression. In a canonical manner, wild-type OdTBK1 significantly phosphorylated interferon regulatory factor 3 (IRF3) but failed when the N-terminal region was truncated. Furthermore, overexpression of OdTBK1 decreased viral proliferation remarkably. Collectively, these data systematically analyzed the characterization and function of OdTBK1, initiating the study of the innate antiviral response of dark sleeper.

## Highlights

- OdTBK1 promotes fish IFNs transcription.
- OdTBK1 phosphorylates IRF3 as a kinase.
- OdTBK1 exhibits a powerful antiviral capacity.

## Introduction

A variety of pattern recognition receptors (PRRs) mediate a host's innate immune response to pathogen invasion; six families have been identified, including Toll-like receptors (TLRs), C-type lectins (CTLs), NOD-like receptors (NLRs), RIG-I-like receptors (RLRs), AIM2-like receptors (ALRs), and OAS-like receptors (OLRs). Among them, RLRs are cytoplasmic sensors of RNA (1). Upon activation, they signal to the mitochondrial antiviral signaling protein (MAVS, also called IPS-1, Cardif, or VISA) (2–4) to form active MAVS polymers and subsequently recruit tumor necrosis factor (TNF) receptor-associated factor (TRAF) family ubiquitin E3 ligases to synthesize polyubiquitin chains, which activates IKK and TANK-binding kinase 1 (TBK1). The recruited kinases then phosphorylate MAVS at its conserved pLxIS motif, and, at the same time, the potential transcription factors interferon regulatory factor (IRF) 3 and IRF7 are phosphorylated by IκB kinase IKKε or TBK1. Thereafter, phosphorylated IRF3 and IRF7 form dimers that accumulate in the nuclei where they bind to target sequences to activate the IFN-β promoter to initiate interferon (IFN) secretion (5).

TBK1 contains an N-terminal kinase, an ubiquitin like domain and two C-terminal coiled-coil domains (6). Recruiting of multiple TBK1 dimers to signaling complexes enables activation-loop swapping of locally clustered TBK1 and results in TBK1 auto-phosphorylation (7). In addition, TBK1 is a serine/threonine protein kinase of the IKK kinase family involved in innate immunity to viral infection by inducing type I IFNs and mediating TANK's ability to activate NF-κB (6, 8).

Upon stimulation by virus structural components, pattern recognition receptors (PRRs) recruit TLR/IL-1R domain-containing adaptor proteins, inducing IFN-β (TRIF), MAVS, and a stimulator of IFN genes (STING; also called MITA, MPYS, and ERIS) to activate TBK1 (9–11). Activated TBK1 phosphorylates IRF3/7, triggers dimerization and nuclear translocation, and leads to formation of active transcriptional complexes that bind to IFN stimulation response elements (ISRE), activating type I IFN gene expression (12). Knockdown assays and gene-targeting studies have shown that TBK1 is essential for type I IFN production in TLR3 and RLR signaling pathways (13–15).

TBK1 has been identified in a variety of fishes. The TBK1 of black carp (*Mylopharyngodon piceus*), named bcTBK1, shows antiviral activity against both Grass Carp Reovirus (GCRV) and Spring Viremia of Carp Virus (SVCV) (16). TBK1 in the large yellow croaker (*Larimichthys crocea*) (named LcTBK1) can interact with an E3 ubiquitin ligase LcNrdp1 to defend against *Cryptocaryon irritans* infection (17). Upon LPS stimulation, TBK1 from grass carp (*Ctenopharyngodon idella*) (CiTBK1) triggers IFN response in an IRF3/IRF7-independent manner (18). In addition, *Danio rerio* TBK1 (DrTBK1) or *Carassius auratus* TBK1 (CaTBK1) can cause the phosphorylation of IRF3/7 (19, 20). Nevertheless, in teleost fish, the identification of the molecular characteristics and mechanisms of TBK1 are still limited.

The dark sleeper (*Odontobutis obscurus*), an important commercial fish species, is widely distributed in the river systems of southeast China (21, 22). In recent years, because of its delicious taste and high nutritional value, the dark sleeper has become a very promising aquaculture species in China. However, some viruses seriously threaten its growth. Better understanding of antiviral immune mechanisms may contribute to the development of management strategies for disease control.

In this study, we report the characterization of *Odontobutis obscurus* (*O. obscura*) TBK1 (OdTBK1). Our findings demonstrate that OdTBK1 is effective for zebrafish and grass carp IFN promoters and significantly induces IFN-stimulated gene (ISG) expression. It can also promote the phosphorylation of IRF3 both in *Danio rerio* (*D. rerio*) and *O. obscura*. OdTBK1 plays a critical role in the antiviral immune defense of fish in promoting the IFN response.

## Materials and Methods

### Cells and Viruses

Epithelioma papulosum cyprinid (EPC, now IDed as fathead minnow) and Grass carp ovary (GCO) cells were maintained at 28°C, 5% CO_2_ in medium 199 (Invitrogen) supplemented with 15% fetal bovine serum (FBS, Invitrogen) (23). Human embryonic kidney (HEK) 293T cells were grown at 37°C, 5% CO_2_ in DMEM medium (Invitrogen) supplemented with 15% FBS. Spring Viremia of Carp Virus (SVCV-741, 10^9^ TCID_50_/ml), a negative-sense ssRNA virus, was propagated in EPC cells until cytopathic effect (CPE) were observed, then the cultured media with cells was harvested and stored at −80°C until use.

### Fish

Healthy dark sleeper (weighing 40 **±** 20 g) purchased from a fish farm were maintained and acclimated to re-circulating tanks (1,000-L, 28 ± 1°C) containing filtered and oxygenated water for at least 2 weeks before experiments. All animal experiments were approved by the Committee on the Ethics of Animal Experiments of the Chinese Academy of Sciences.

### Amplification of OdTBK1 and Plasmid Construction

Rapid amplification of cDNA ends (RACE) was carried out using the 5′ RACE system (Invitrogen) and BD SMART^TM^ RACE cDNA amplification kit (BD Biosciences Clontech) according to the manufacturer's instruction. The first strand cDNA synthesis and RACE were performed on liver-derived RNA. To obtain the 3′ unknown region, primer pairs OdTBK1F4a/APT and OdTBK1F5a/AP (Table 1), were used for the primary PCR and the nested PCR, respectively. The amplified PCR product was cloned and sequenced as described above. Similarly, the 5′ end of OdTBK1 was obtained by nested PCR using primer pairs OdTBK1R2a/APG and OdTBK1R3a/AP (Table 1). The full-length cDNA sequence was confirmed by sequencing the PCR product amplified by primers OdTBK1-FP and OdTBK1-RP (Table 1) within the predicted 5′ and 3′ untranslated regions, respectively. The open reading frame (ORF) of OdTBK1 was subcloned into pcDNA3.1(+) (Invitrogen), pCMV-Myc (Clontech) and pCMV-Tag2C (Clontech), respectively. For subcellular localization, the ORF of OdTBK1 was inserted into pEGFP-N3 (Clontech) vector. The ORFs of zebrafish IRF3 (NM_001143904) was subcloned into pCMV-HA (Clontech) and pCMV-Myc vector. The primers used to amplify the N-terminal absent OdTBK1 sequence were OdTBK1-ΔN-FP and OdTBK1-RP (Table 1). It was also subcloned into pCMV-Tag2C and pCMV-Myc vector. The fmIFN promoter was obtained from NCBI database (HE856618.1) and cloned into pGL3-Basic luciferase reporter vector (Promega). The plasmids containing DrIFNϕ1pro-Luc, DrIFNϕ3pro-Luc, gcIFN1, and ISRE-Luc in pGL3-Basic luciferase reporter vectors were constructed as described previously (24, 25). All constructs were confirmed by DNA sequencing.

**Table 1:** Primers used in this study.

**Name**	**Sequences (5^**′**^ → 3^**′**^)**	**Purpose**
OdTBK1-F1a	CCTCGCCAACATCCTGGAGGCCG	RACE
OPTBK1-R1a	CGTACAGCAGCTCCTGGTTGTG	
OdTBK1-R2a	GGTGAACAATGCCATACTCTC	
OdTBK1-R3a	TCGTCCTCTAGCTCTCTGG	
OdTBK1-F4a	GGTGAGATCTCGGACATGC	
OdTBK1-F5a	GCATGCTGACTGATACCTGG	
APT	CCAGACTCGTGGCTGATGCATTTTTTTTTTTTTTTTTTTV	
APG	CCAGACTCGTGGCTGATGCAGGGGGGGGGGGGGGGGV	
AP	CCAGACTCGTGGCTGATGCA	
OdTBK1-FP	ATGCAGAGCACCACTAACTAC	Eukaryotic expression
OdTBK1-RP	TTAACCCCTCAGTCCTCCGTCC	
OdTBK1-ΔN-FP	CCGGAATTCCGATGCAGAGCACCACTAACTACCTTACAGTGGCACTGTTTCAGGAC	
qPCR-OdTBK1-FP	GGAGACCTGTATGCTGTC	Real-time PCR
qPCR-OdTBK1-RP	CAATACTCCATCACCAGC	
qPCR-IFN-EPC-FP	ATGAAAACTCAAATGTGGACGTA	
qPCR-IFN-EPC-RP	GATAGTTTCCACCCATTTCCTTAA	
qPCR-VIG1-EPC-FP	AGCGAGGCTTACGACTTCTG	
qPCR-VIG1-EPC-RP	GCACCAACTCTCCCAGAAAA	
qPCR-MAVS-EPC-FP	GAATGTCCCTGTCCGAGAAA	
qPCR-MAVS-EPC-RP	TCTGAACATGCTCGTTTGCAG	
qPCR-β-actin-FP	CACTGTGCCCATCTACGAG	
qPCR-β-actin-RP	CCATCTCCTGCTCGAAGTC	
qPCR-Od-β-actin-FP	CTCTTCCAGCCATCCTTCCT	
qPCR-Od-β-actin-RP	TCAGGTGGGGCAATGATCTT	

### Bioinformatics Analysis

The phylogenetic tree was constructed with the Neighbor-joining method (NJ) by MEGA7 program which was bootstrapped 500 times. All gene sequences used in this study were derived from GenBank. Multiple alignments were accomplished using GENE.DOC.

### Transient Transfection and Virus Infection

Transient transfections were performed in EPC cells or GCO cells seeded in 6-well or 24-well plates by using X-tremeGENE HP DNA Transfection Reagent (Roche) according to the manufacturer's protocol. And the transfection efficiency of EPC cells or GCO cells is nearly 50%. To confirm the antiviral response of OdTBK1, EPC cells were seeded in 24-well plates overnight and transfected with 0.5 μg OdTBK1-Myc or the pCMV-Myc vector separately. At 24 h post-transfection, the EPC cells were infected with SVCV at a multiplicity of infection (MOI = 0.01) and incubated at 28°C. The supernatant aliquots were then harvested to detect the virus titers at 48 h post infection, using the standard 50% tissue culture infection dose (TCID_50_) method. The cell monolayers were washed with PBS, fixed with 4% paraformaldehyde (PFA) for 1 h, and stained with 0.05% crystal violet overnight, and observed for the cytopathic effect (CPE). For virus titration, 200 μl of culture medium were collected at 48 h post-infection, and used for plaque assay. The supernatants were subjected to 3-fold serial dilutions and then added (100 μl) onto a monolayer of EPC cells cultured in a 96-well plate. After 48 or 72 h, the medium was removed and the cells were washed with PBS, fixed by 4% PFA and stained with 1% crystal violet. The virus titer was expressed as 50% tissue culture infective dose (TCID_50_/ml). Results are representative of three independent experiments.

### Luciferase Activity Assay

EPC cells were seeded in 24-well plates, and 24 h later co-transfected with 250 ng luciferase reporter plasmid (DrIFNϕ1pro-Luc, DrIFNϕ3pro-Luc, ISRE-Luc, or fmIFN-Luc) and 250 ng OdTBK1-Myc or pCMV-Myc and 50 ng *Renilla* luciferase internal control vector (pRL-TK, Promega). Empty vector pCMV-Myc was used to maintain equivalent amounts of DNA in each well. GCO cells were used to detect ISRE-Luc and gcIFN1-Luc. At 24 h post-transfection, the cells were washed in PBS and lysed for measuring luciferase activity by Dual-Luciferase Reporter Assay System, according to the manufacturer's instructions (Promega). Firefly luciferase activities were normalized on the basis of *Renilla* luciferase activity. The results were representative of more than three independent experiments, each performed in triplicate.

### Fluorescence Microscopy

EPC cells were plated onto coverslips in 6-well plates and transfected with indicated plasmids for 24 h. The experimental group was stimulated with the viral analog polyinosinic-polycytidylic acid (poly I:C) 12 h before the photo was taken. Then the cells were washed twice with PBS and fixed with 4% PFA for 1 h. After draining the fixative, the cells were stained with DAPI (C1006, Beyotime) for 5 min in dark at room temperature. Finally, the coverslips were washed and observed with a Leica confocal microscope under a × 63 oil immersion objective (SP8; Leica Microsystems).

### RNA Extraction, Reverse Transcription, and Quantitative Real-Time PCR

The total RNA from brain, pituitarium, eye, gill, heart, liver, muscle, spleen, head kidney, and ovary were extracted from three apparently healthy dark sleepers to detect the expression of OdTBK1.

Total RNAs of EPC cells and tissues were extracted by Trizol reagent (Invitrogen). RNase-free DNase is used specifically for RNA purification by removing all contaminating genomic DNA. The first-strand cDNA was synthesized by using a GoScript Reverse Transcription System (Promega) according to the manufacturer's instructions. Quantitative real-time PCR (qPCR) was performed with Fast SYBR Green master mix (BioRad) on a CFX96 Real-Time System (BioRad). PCR conditions were as follows: 95°C for 5 min, then 40 cycles of 95°C for 20 s, 60°C for 20 s, 72°C for 20 s. and the β-actin primers were used as internal control. The specificity of the PCR amplification for all primer sets was verified from the dissociation curves. The identity of each PCR products was confirmed by dideoxy-mediated chain termination sequencing at Wuhan TSINGKE Biological Technology Inc. The relative fold changes were calculated by comparison to the corresponding controls using the 2^−Δ*ΔCt*^ method. Three independent experiments were conducted for statistical analysis. The total RNA extracted from the tissue of liver also treated as cells.

### Co-IP Assay

For transient-transfection and Co-IP experiments, HEK 293T cells were used instead of EPC cells due to the superhigh transfection efficiency of HEK 293T cells. Cells seeded into 10-cm^2^ dishes overnight were transfected with a total of 10 μg the indicated plasmids. At 24 h post transfection, the medium was removed carefully, and the cell monolayer was washed twice with 10 ml ice-cold phosphate-buffered saline (PBS). The cells were then lysed in 1.0 ml radioimmunoprecipitation (RIPA) lysis buffer (1% NP-40, 50 mM Tris-HCl [pH 7.5], 150 mM NaCl, 1 mM EDTA, 1 mM NaF, 1 mM sodium orthovanadate [Na_3_VO_4_], 1 mM phenylmethylsulfonyl fluoride [PMSF], 0.25% sodium deoxycholate) containing a protease inhibitor cocktail (Sigma-Aldrich) at 4°C for 1 h on a rocker platform. The cellular debris was removed by centrifugation at 12,000 × *g* for 15 min at 4°C. The supernatant was transferred to a fresh tube and incubated with 20 μl anti-Flag affinity gel (Sigma-Aldrich) overnight at 4°C with constant agitation. These samples were further analyzed by Immunoblotting (IB). Immunoprecipitated proteins were collected by centrifugation at 5,000 × *g* for 1 min at 4°C, washed three times with lysis buffer, and resuspended in 100 μl 5 × SDS sample buffer. The immunoprecipitates and whole-cell lysates were analyzed by IB with the indicated Abs.

### *In vitro* Protein Dephosphorylation Assay

Transfected HEK 293T cells were lysed as described above, except that the phosphatase inhibitors (Na_3_VO_4_ and EDTA) were omitted from the lysis buffer. Protein dephosphorylation was carried out in 100 μl reaction mixtures consisting of 100 μg of cell protein and 10 U of CIP (Sigma-Aldrich). The reaction mixtures were incubated at 37°C for 1 h, followed by Western blotting analysis.

### Immunoblot Analysis

Whole cells were lysed in radioimmunoprecipitation (RIPA) lysis buffer [1% NP-40, 50 mM Tris-HCl (pH 7.5), 150 mM NaCl, 1 mM EDTA, 1 mM NaF, 1 mM sodium orthovanadate, 1 mM phenyl-methylsulfonyl fluoride, and 0.25% sodium deoxycholate] containing protease inhibitor mixture (Sigma-Aldrich). The Bradford method was used for estimation of protein concentration of cell lysates. The equivalent amount of proteins (10 μg) in different groups were separated by 10% SDS-PAGE and transferred to PVDF membrane (Bio-Rad). The membranes were blocked for 1 h at room temperature in TBST buffer (25 mM Tris-HCl, 150 mM NaCl, 0.1% Tween-20, pH 7.5) containing 5% nonfat dry milk, probed with indicated primary antibodies (Abs) at an appropriate dilution overnight at 4°C, washed three times with TBST and then incubated with secondary Abs for 1 h at room temperature. After additional three washes with TBST, the membranes were stained with Immobilon TM Western Chemiluminescent HRP Substrate (Millipore) and detected using an Image Quant LAS4000 system (GE Healthcare). Abs were diluted as follows: anti-Myc (Santa Cruz Biotechnology) at 1:2000, HRP-conjugated anti-mouse IgG (Thermo Scientific) at 1:5000. The results were the representative of three independent experiments.

### Statistics Analysis

The results are expressed as mean ± SEM. Data were analyzed using a Student's unpaired *t-*tests. A *p* < 0.05 was considered to be statistically significant.

## Results

### Molecular Cloning and Phylogenetic Analysis of OdTBK1

**Figure 1:**
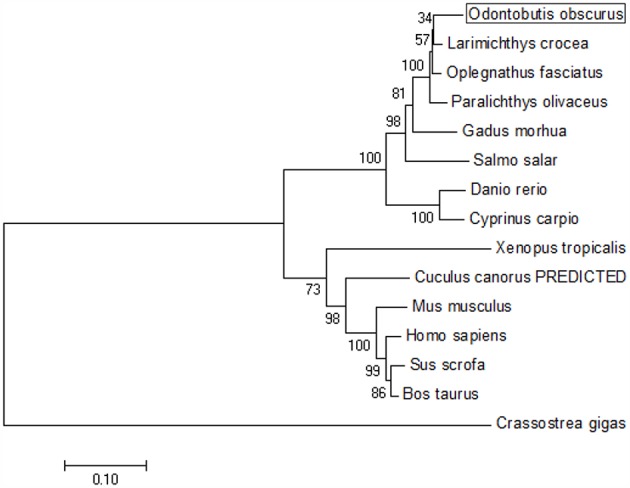
Phylogenetic tree of various TBK1 members. The tree was depicted on the overall sequences by neighbor-joining method. The 0.1 scale indicates the genetic distance. Accession numbers of TBK1 amino acid sequences are as follows: *Odontobutis obscura* (dark sleeper), MK637832; *Oplegnathus fasciatus* (Stone bream), AHX37216.1; *Paralichthys olivaceus* (bastard halibut), AGL54166.1; *Larimichthys crocea* (large yellow croaker), AKM77645.1; *Salmo salar* (Atlantic salmon), AEA42006.1; *Gadus morhua* (Atlantic cod), ADL60136.1; *Danio rerio* (zebra fish), NP_001038213.2; *Cyprinus carpio* (koi), ADZ55455.1; *Xenopus tropicalis* (Western clawed frog), NP_001135652.1; *Crassostrea gigas* (Thunberg), APX43288.1; *Cuculus canorus* (Cuckoo), XP_009554581.1; *Mus musculus* (Mouse), AAF05990.1; *Sus scrofa* (Pig), NP_001098762.1; *Bos taurus* (Bovine), NP_001179684.1; *Homo sapiens* (Human), AAF05989.1.

**Figure 2:**
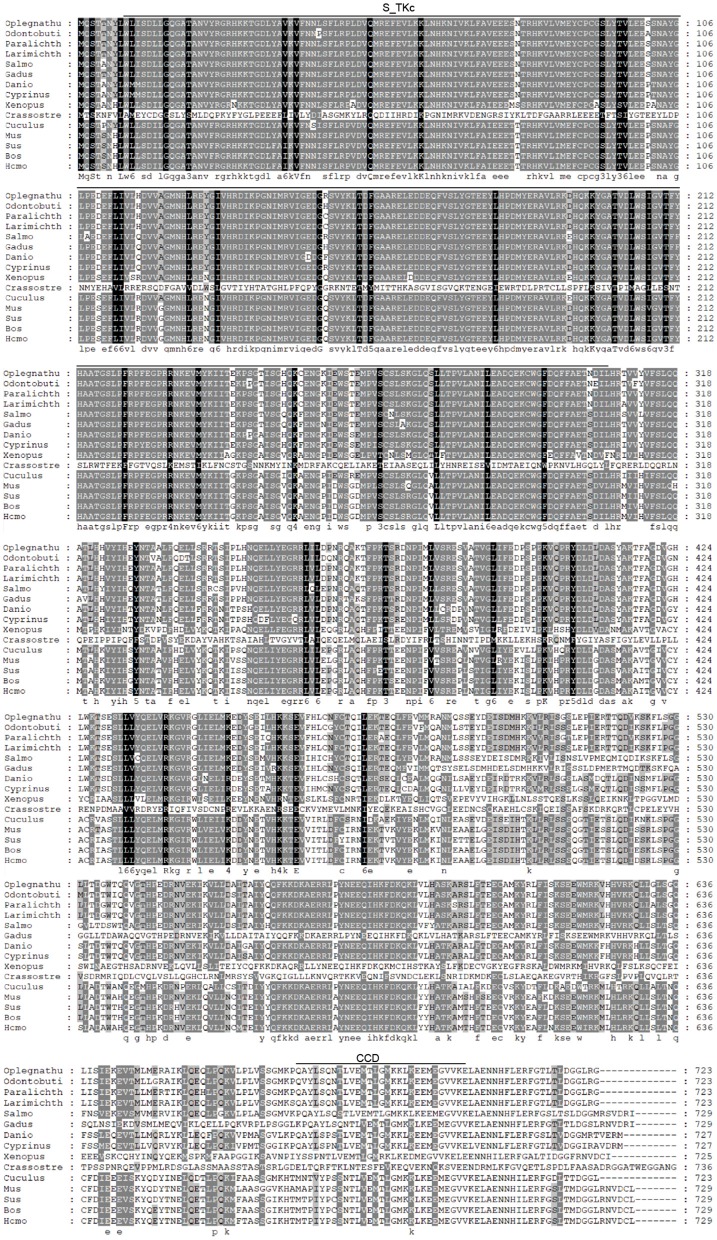
Sequence alignment of the amino acid sequence of TBK1 from several species was carried out by Gene Doc. Conserved N-terminal Serine/Threonine protein kinases catalytic domain (S_TKc) was showed in the black box, C-terminal coiled coil domain (CCD) was showed in the gray box.

The coding sequence (CDS) of OdTBK1 was obtained by RACE-PCR. The full-length ORF of OdTBK1 is 2,172 bp, encoding 723 amino acids through initial sequence analysis of OdTBK1 prediction. The SMART program predicted that there were Serine/Threonine protein kinases catalytic domain (S_TKc) in N terminus (9-306) and coiled coil domain (CCD) in C terminus (678-704). To investigate the homology of OdTBK1, phylogenetic analyses of TBK1 with several species were conducted by MEGA7 based on the CDS sequences; these members could be divided into mammals, amphibians, mollusks, and fish (Figure 1). OdTBK1 showed high homology with the TBK1 of *L. crocea*. Additionally, OdTBK1 and the TBK1 homologs of *D. rerio* and *G. morhua* shared similar positions on the homology. Meanwhile, multiple alignments of OdTBK1 to the corresponding sequences of other species showed that the N-terminal kinases catalytic domains of TBK from the teleost were conserved (Figure 2).

### Tissue-Specific Expression Pattern of OdTBK1

**Figure 3:**
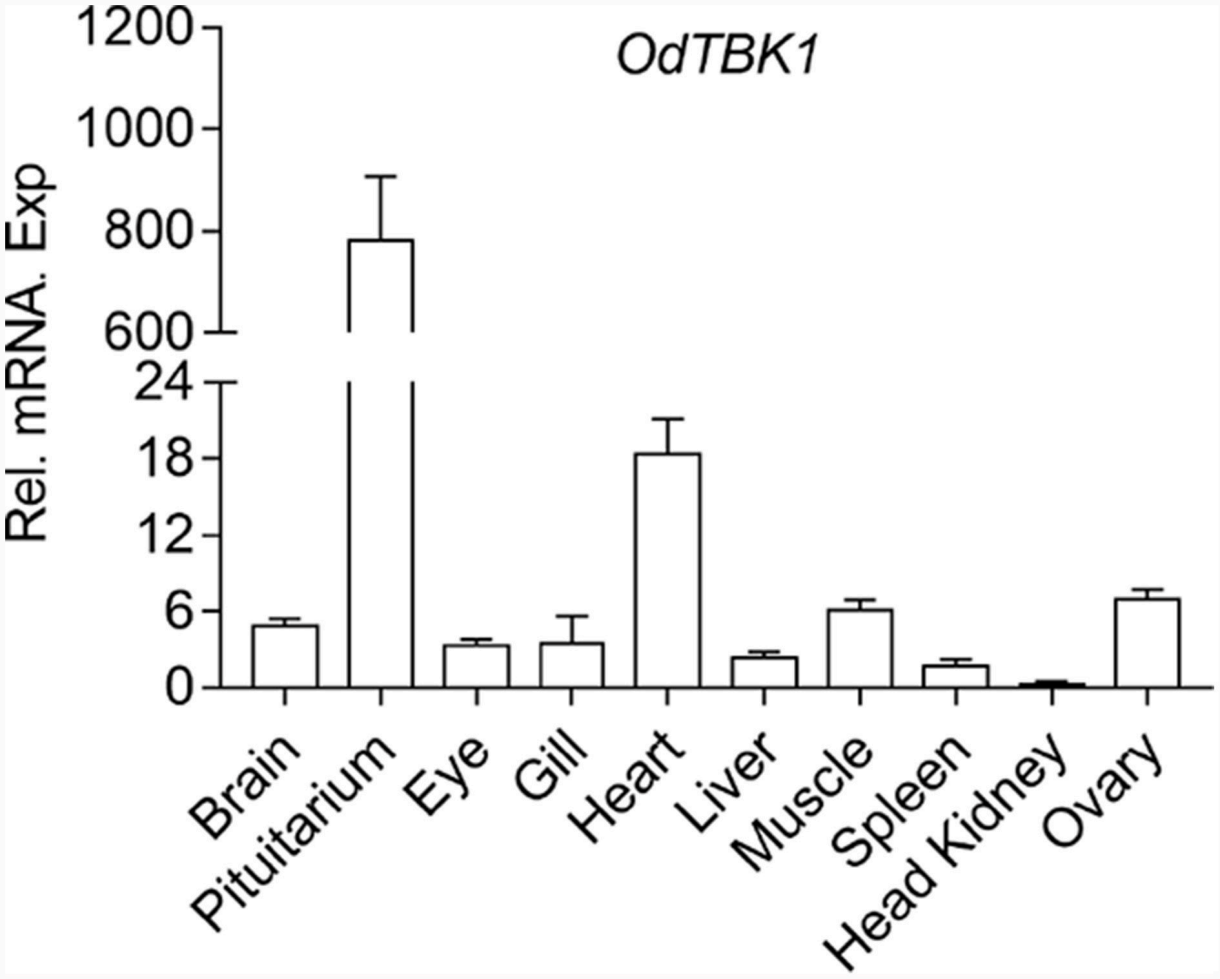
Expression analysis of OdTBK1 in different tissues. Total RNAs of various tissues in dark sleeper were extracted to examine the transcripts of OdTBK1, β*-actin* was used as an internal control for normalization to give relative expression. The expression levels in the other tissues are shown as fold induction compared with the head kidney (for which their relative expressions were set to 1). Error bars represents the means ± SEM (*n* = 3), and the experiments were repeated three time with similar results.

To investigate the mRNA level of OdTBK1 *in vivo*, total RNA was isolated from brain, pituitarium, eye, gill, heart, liver, muscle, spleen, kidney, and ovary of the black sleeper separately. The tissue distribution pattern of OdTBK1 was monitored by qRT-PCR. Transcripts of OdTBK1 were detected in all ten tissues: the highest level of OdTBK1 was observed in pituitarium, while the lowest level of OdTBK1 appeared in the head kidney. Generally, the expression levels of OdTBK1 were basically the same across tissues (Figure 3). These data demonstrated that OdTBK1 was ubiquitously expressed in the tissues of the dark sleeper.

### OdTBK1-Mediated IFNs and ISRE Activation

**Figure 4:**
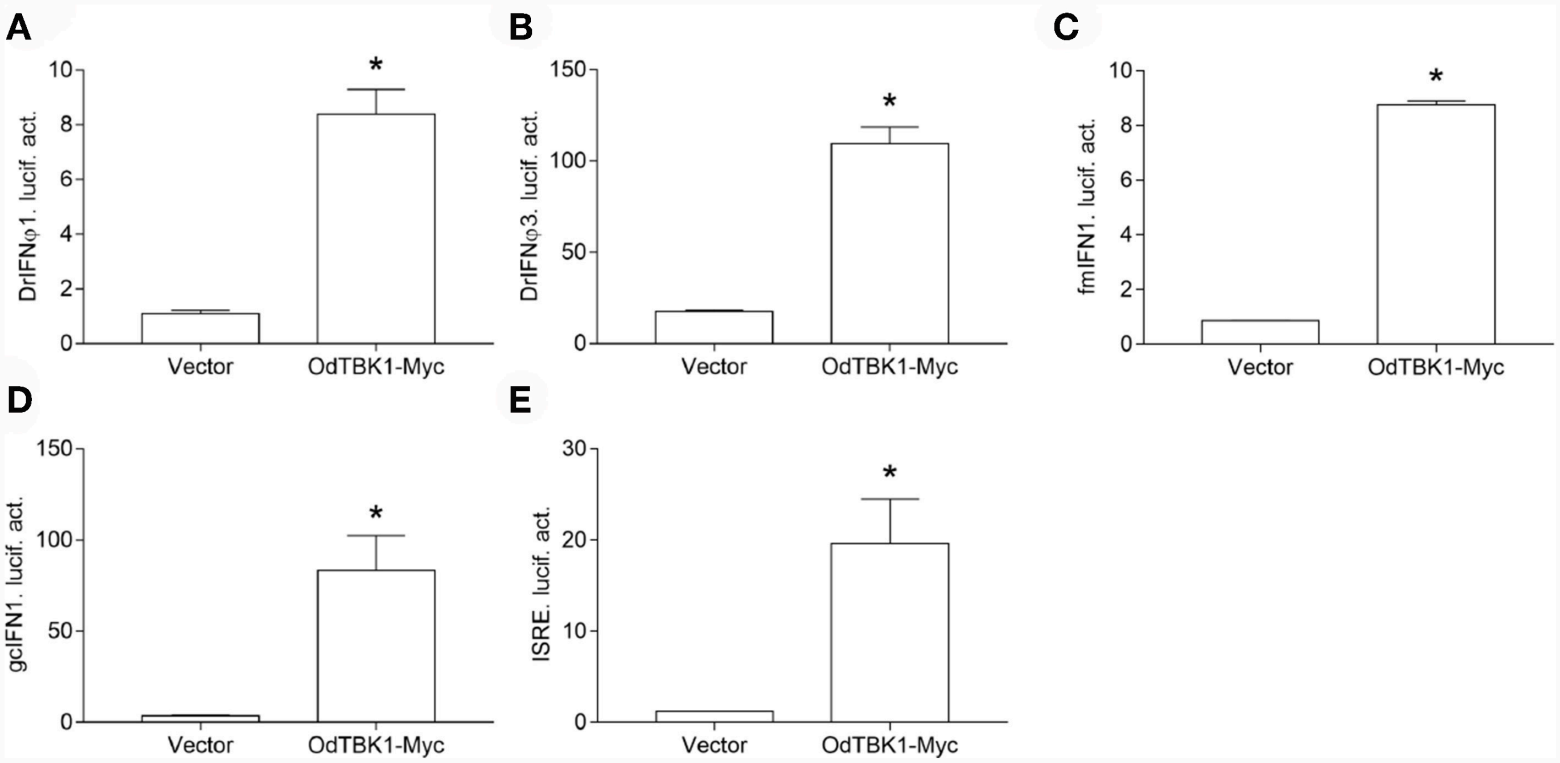
**(A–E)** Activation of DrIFNϕ1, DrIFNϕ3 and ISRE promoters by overexpression of OdTBK1. EPC cells were seeded on 24-well-plates overnight and co-transfected with pCMV-Myc or OdTBK1-Myc and DrIFNϕ1pro-Luc **(A)**, DrIFNϕ3pro-Luc **(B)**, fmIFNpro-Luc **(C)**. GCO cells were treated at the same way and co-transfected with pCMV-Myc or OdTBK1-Myc and gcIFN1pro-Luc **(D)**, ISRE-Luc **(E)**. The luciferase assays were performed 24 h after transfection. The promoter activity is presented as relative light units normalized to *Renilla* luciferase activity. Error bars represents the means ± SEM (*n* = 3), and the experiments were repeated three time with similar results. Asterisks indicate significant differences from control (^*^p < 0.05).

TBK1 is a crucial factor involved in type I IFN response. To clarify whether OdTBK1 activates IFN production, the luciferase report gene assay was employed in the following manner. Co-transfection of the OdTBK1 and IFN promoters occurred in EPC cells, and the activities of these promoters were measured 24 h later. According to the results, OdTBK1 significantly upregulated the activation of the DrIFNϕ1 promoter up to 7.6-fold compared with the empty vector control group (Figure 4A). Similarly, the activation of the DrIFNϕ3 promoter increased by 6.3-fold compared with the control group (Figure 4B). The activation of the fmIFN promoter was also measured, and it was significantly induced about 20-fold compared with the empty vector control group (Figure 4C). Then, ISRE was co-transfected with OdTBK1 in GCO cells to monitor the capacity of OdTBK1 in another fish cell line. Consistent with the result above, ISRE was significantly enhanced 24-fold by OdTBK1 overexpression (Figure 4D). Finally, GcIFN1 was co-transfected with OdTBK1 in the same way, and its activity was also upregulated about 16-fold (Figure 4E). These results demonstrated that OdTBK1 conservatively activated the IFN promoters from different fishes.

### Cytoplasm Localization of OdTBK1

**Figure 5:**
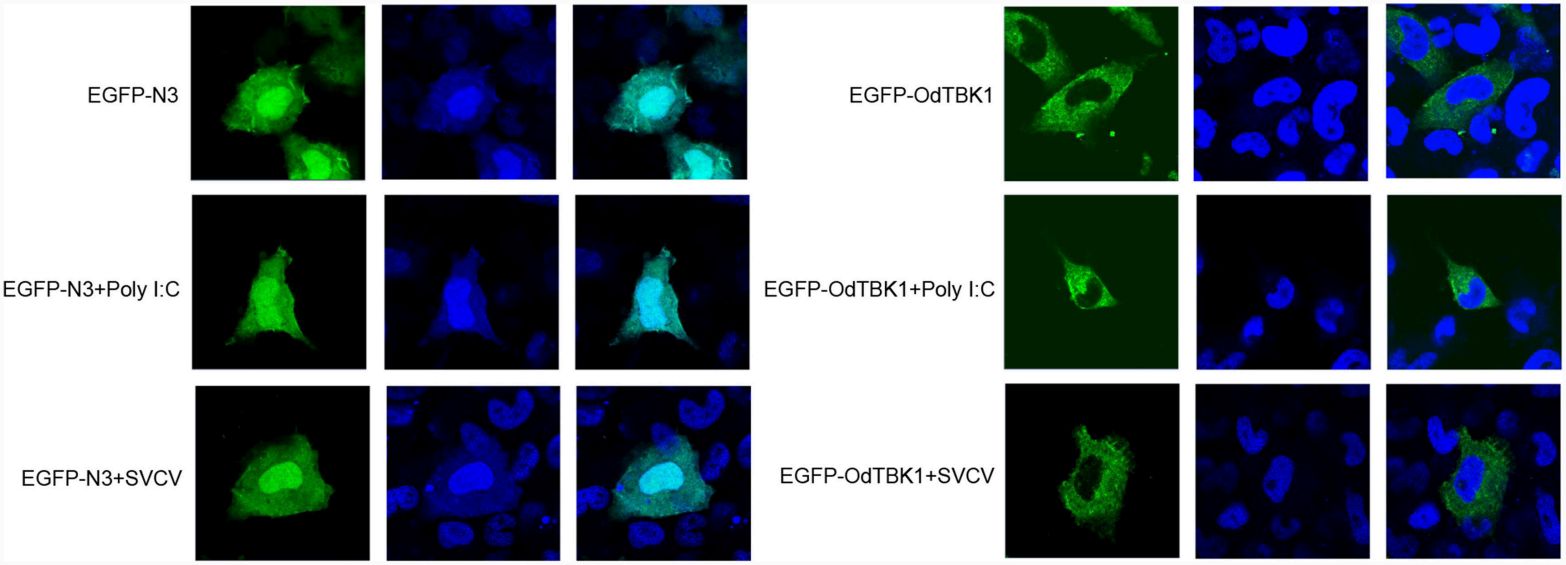
Subcellular localization of OdTBK1. EPC cells seeded onto microscopy cover glass in 6-well plates were transfected with 2 μg EGFP-OdTBK1 or the empty vector. After 24 h, the cells were untreated (null) or transfected with 2 μg poly (I:C) or infected with 4 μl SVCV for 12 h, then the cells were fixed and subjected to confocal microscopy analysis. Green staining represents the OdTBK1 protein signal, and blue staining indicates the nucleus region (original magnification, ×63; oil immersion objective). Bar, 10 μm. All experiments were repeated at least three times, with similar results.

Fluorescence microscopy was used to investigate the subcellular localization of OdTBK1 with or without I:C stimulation or SVCV infection. Poly I:C, an RNA virus mimic, can induce IFN expression significantly. EPC cells transfected with EGFP-N3 were used as a control group. The fluorescent signals of EGFP-N3 were distributed in the cytosol and the nucleus with or without poly I:C stimulation or SVCV infection. Compared with the controls, without stimulation, the green signals of OdTBK1 were detected in the cytosol, with none in the nucleus. After poly I:C stimulation or SVCV infection, green fluorescent signals were also detected only in the cytosol (Figure 5). These data suggested that OdTBK1 was localized in the cytoplasm.

### OdTBK1 Acted as a Kinase to Phosphorylate IRF3

**Figure 6:**
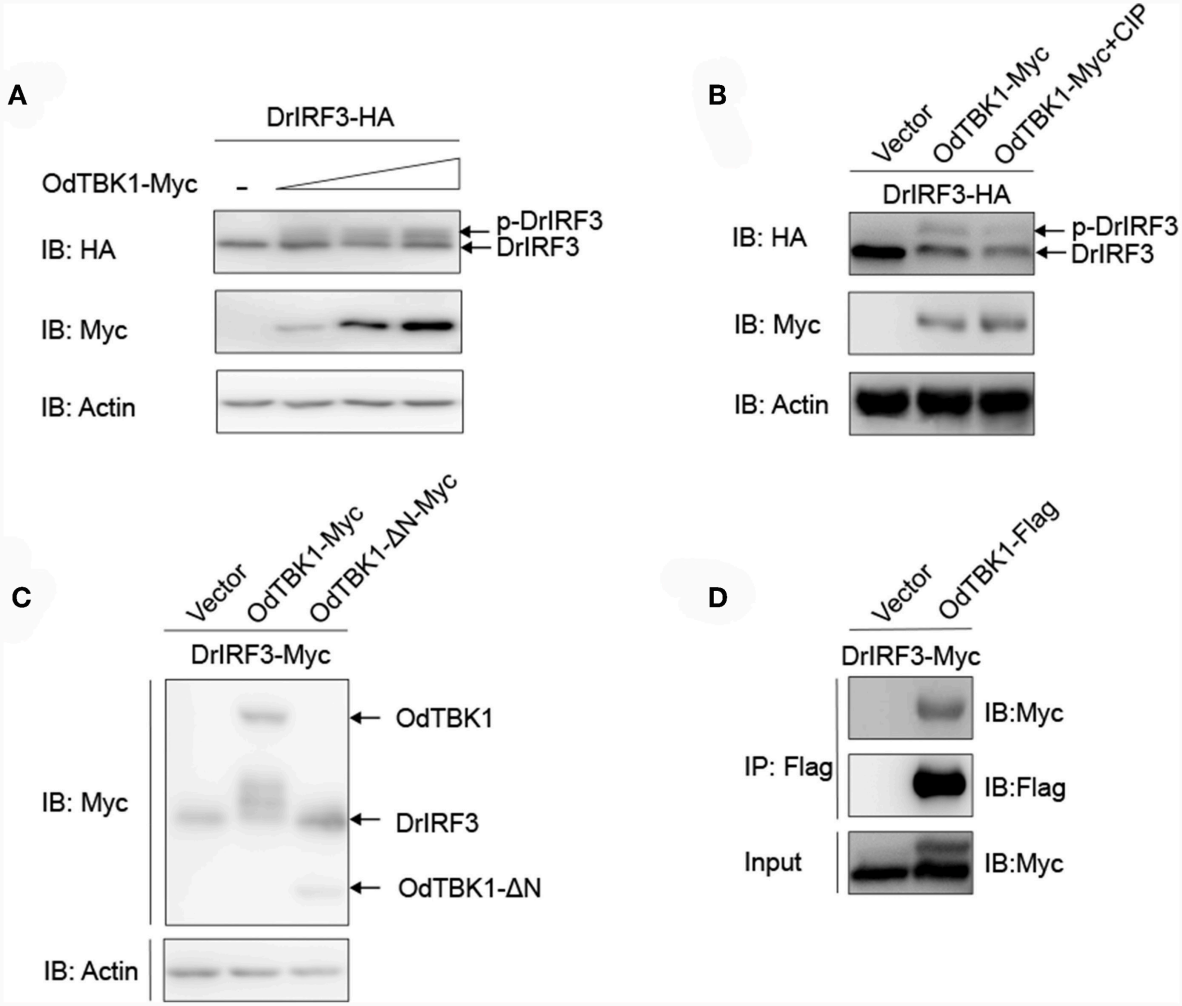
IRF3 was phosphorylated by OdTBK1. **(A)** Overexpression of OdTBK1 induces the phosphorylation of DrIRF3 in a dose-dependent manner. HEK 293T cells were seeded in six-well plates overnight and co-transfected with 1 μg DrIRF3-HA (attachment of a HA tag to the N terminus of DrIRF3) and 1 μg empty vector, or OdTBK1-Myc (0.25, 0.5 and 1 μg, respectively) for 24 h. The cell lysates were subjected to IB with the anti-HA, anti-Myc, and anti-β-actin Abs. **(B)** OdTBK1 mediates the phosphorylation of DrIRF3. HEK 293T cells were seeded in six-well plates overnight and co-transfected with 1 μg DrIRF3-HA and 1 μg empty vector or OdTBK1-Myc for 24 h. The cell lysates (100 μg) were treated with or without CIP (10 U) for 40 min at 37°C. The lysates were then subjected to IB with the anti-HA, anti-Myc, and anti-β-actin Abs. **(C)** IRF3 was phosphorylated by the N terminus of OdTBK1. HEK 293T cells were seeded in six-well plates overnight and transfected with the indicated plasmids (1 μg each) for 24 h. The cell lysates were subjected to IB with the anti-Myc and anti-β-actin Abs. **(D)** OdTBK1 associates with DrIRF3. HEK 293T cells seeded into 10-cm^2^ dishes were transfected with the indicated plasmids (10 μg each). After 24 h, cell lysates were immunoprecipitated (IP) with anti-Flag affinity gel. The immunoprecipitates and cell lysates were then analyzed by IB with anti-Myc and anti-Flag Abs, respectively. All experiments were repeated at least three times with similar results.

As mentioned earlier, IRF3 is a crucial downstream signaling molecule of TBK1. After co-transfection of zebrafish IRF3 (DrIRF3) and OdTBK1, cells were lysed for immunoblotting. Compared with the signal band of DrIRF3 in the control group, band shifts occurred through stimulation with OdTBK1 (Figure 6A). To confirm whether the shifted bands represented phosphorylated IRF3, a dephosphorylation assay was performed *in vitro*. The shifted bands partially disappeared after being treated with calf intestinal phosphatase (CIP), demonstrating that DrIRF3 can be phosphorylated by OdTBK1 (Figure 6B). To further characterize this process, a truncated OdTBK1 mutant was generated to identify the functional domain that regulates the phosphorylation of DrIRF3. As shown in Figure 6C, the mutant lacking the kinase domain (OdTBK1-ΔN) failed to phosphorylate DrIRF3, suggesting that this domain was indispensable for its kinase activity (Figure 6C). In addition, to validate the association between OdTBK1 and DrIRF3, we transfected expression plasmids for DrIRF3-Myc and OdTBK1-Flag into HEK 293T cells and performed Co-IP assays. The results indicate that OdTBK1 interacted with DrIRF3 (Figure 6D) and was involved in phosphorylation of DrIRF3 via its functional kinase domain.

### OdTBK1 Induced Downstream ISGs Expression

**Figure 7:**
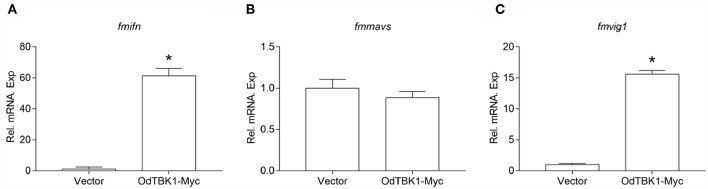
Overexpression of OdTBK1 induces the expressions of ISGs in EPC cells. **(A–C)** qPCR detection of the transcript levels of ISGs in OdTBK1-overexpressing cells. EPC cells seeded in 6-well plates overnight were transfected with 1.5μg OdIRF3-Myc or empty vector for 24 h. Then total RNAs were extracted to examine the transcripts of *ifn* **(A)**, *mavs* **(B)**, and *vig1* **(C)** by qPCR. β*-actin* was used as an internal control for normalization and the relative expression is represented as fold induction relative to the expression level in control cells (set to 1). Error bars represents the means ± SEM (*n* = 3), and the experiments were repeated three time with similar results. Asterisks indicate significant differences from control (^*^p < 0.05).

OdTBK1 significantly phosphorylates IRF3, which is a crucial transcriptional factor of IFN, to activate ISGs expression. Therefore, the regulation activity of OdTBK1 was monitored. EPC cells were transfected with OdTBK1 and total RNA was extracted for reverse transcription. qRT-PCR was employed to detect several genes involved in IFN-mediated pathways, such as *ifn, mavs*, and *vig1* (26). The transcription levels of these ISGs increased significantly compared with the control group. The mRNA level of *ifn* was increased by 61-fold compared with the control group, and that of *vig1* was increased by 15-fold (Figures 7A–C). These data indicated that OdTBK1 was capable of activating ISG transcription.

### Antiviral Activity of OdTBK1

**Figure 8:**
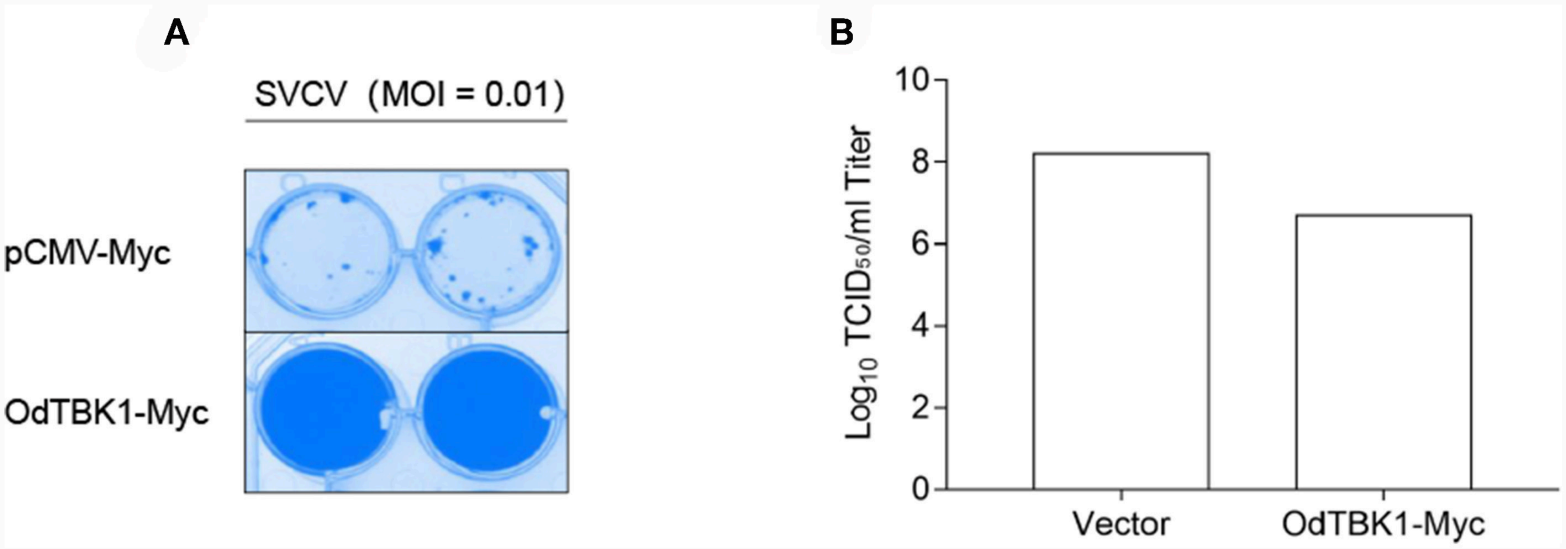
Antiviral assay on OdTBK1. **(A,B)** EPC cells seeded in 24-well plates overnight were transfected with 0.5 μg OdTBK1-Myc or pCMV-Myc vector. At 24 h after transfection, the EPC cells were infected with SVCV (MOI = 0.01) per well and incubated at 28°C. The culture media was then harvested to detect the virus titers at 48 h post infection. **(A)** The cell monolayers were fixed with 4% PFA for 1 h and stained with 1% crystal violet overnight. **(B)** The viral titers of the supernatants were determined by TCID_50_ assays on EPC cells. The experiments were performed for three times with similar results.

Finally, to investigate the role of OdTBK1 in host antiviral immunity, EPC cells were transfected with OdTBK1 and the pCMV-Myc group was used as a control group. These two groups were separately infected with identical titers of SVCV at 24 h post-transfection. At 36 h after the infection, apparent CPE was observed in the control cells, while the CPE of the OdTBK1 group was obviously reduced (Figure 8A). Measurement of the virus titers of the OdTBK1 and control groups suggested that it was reduced over 30-fold in the OdTBK1-overexpressing cells compared with the control cells (Figure 8B). The data above demonstrated that OdTBK1 enhanced the antiviral ability of cells against the virus, which indicated that OdTBK1 participated as a crucial factor in the host antiviral innate immune response.

## Discussion

As a key positive IFN regulator, TBK1 has been extensively studied in mammals, whereas studies of teleost TBK1 are rare. In this study, we describe a fish TBK1 isolated from *O. obscura* and its kinase function in inducing IFN expression and phosphorylating IRF3. These data demonstrate that TBK1 is conserved in both lower and higher vertebrates.

In our study, phylogentic analysis of the TBK1 amino acid sequences revealed that dark sleeper TBK1 is closely related to that of other fish and is likely an ortholog of mammalian TBK1s. This is consistent with TBK1 being the crucial kinase in IFN response, an essential and pivotal part of antiviral defense. In addition, other RLR factors such as MAVS, MITA, and IRF3 are conserved and functional in fish. Generally, IFN production activated by the RLR axis is necessary for both fish and mammal antiviral processes.

The tissue expression pattern indicated that OdTBK1 was highest in the pituitary, but relatively low in the spleen and head kidney. This is interesting because the pituitary participates in the formation of the hypothalamic–pituitary–interrenal axis for regulating the metabolism, immune response, and growth of fish generally (27, 28). The lower expression level of OdTBK1 in the head kidney and spleen is probably because OdTBK1 is not necessary in immune-relevant tissues of healthy fish. Also, OdTBK1 nay not be involved in adaptive immune processes.

In mammals, the induction of IFN and downstream ISGs symbolizes antiviral post infection response (29, 30). Similar to this, our study revealed that OdTBK1 exhibits a powerful effect on zebrafish and grass carp IFN promoter activation. Overexpression of OdTBK1 up-regulates the expression of *fmifn* and *fmvig1*, but not of *fmmavs*. MAVS belongs to the IFN-related gene and recruits TBK1/IKKε by TRAFs, which means that MAVS is not the TBK1 downstream effector protein (31). Furthermore, fish MAVS has been reported to contribute to IFN antiviral immunity upstream of TBK1 and IRF3/7 (32). These findings explain why TBK1 cannot directly up-regulate the expression of *fmmavs*.

OdTBK1 was located in the cytoplasm with or without poly I:C and SVCV, indicating that TBK1 activates downstream signal molecules from the cytoplasm and that the cytoplasm is where TBK1 is regulated by upstream molecules. After being stimulated by TBK1, dimerized and phosphorylated IRF3 transfers from the cytoplasm to the nucleus, where it binds to ISRE motifs to initiate the transcription of target genes, including IFN and other ISGs (33). As a critical kinase involved in antiviral immunity, TBK1 activity can be regulated in a variety of ways, such as phosphorylation, ubiquitination, and kinase activity modulation. These modifications occur mainly in the cytoplasm. For instance, E3 ubiquitin ligase TRAF3 mediates lysine 63 (K63)-linked polyubiquitination of TBK1 and facilitates its activation in the cytoplasm (34). These data support the cytoplasmic localization of TBK1 as responsible for its activation and function.

Research has indicated that OdTBK1 exhibited kinase activity that induced the phosphorylation of DrIRF3. We have characterized OdIRF3, but do not yet know whether OdIRF3 can be phosphorylated by OdTBK1. The protein kinase domain of OdTBK1 was indispensable for its kinase activity; the dominant negative mutant TBK1-K38M of human or crucian carp is able to effectively block this activity (20, 33). Future studies are needed to construct the mutant OdTBK1-K38M to determine conservation of the kinase functional site.

In summary, we have identified and characterized OdTBK1 and verified the partial function of TBK1 in host antiviral innate immunity. More research is still needed to understand the molecular mechanisms behind the biological functions and regulation of the TBK1-mediated signaling pathway and to gain insight into the potential role of OdTBK1 in fish.

## Author Contributions

SL conceived and designed the experiments. SL, JC, ZL, LL, PL, and X-YL performed the experiments and analyzed the data. SL, JC, and ZC wrote the manuscript. All authors reviewed the manuscript.

### Conflict of Interest Statement

The authors declare that the research was conducted in the absence of any commercial or financial relationships that could be construed as a potential conflict of interest.

## References

[1] Loo YM Gale M Jr. Immune signaling by RIG-I-like receptors. Immunity. (2011) 34:680–92. 10.1016/j.immuni.2011.05.003 21616437 PMC3177755

[2] He TS Chen T Wang DD Xu LG HAUS8 regulates RLR-VISA antiviral signaling positively by targeting VISA. Mol Med Rep. (2018) 18:2458–66. 10.3892/mmr.2018.9171 29916539

[3] Zhang P Li Y Xia J He J Pu J Xie J. IPS-1 plays an essential role in dsRNA-induced stress granule formation by interacting with PKR and promoting its activation. J Cell Sci. (2014) 127 (Pt. 11):2471–82. 10.1242/jcs.139626 24659800

[4] Haynes LD Verma S McDonald B Wu R Tacke R Nowyhed HN. Cardif (MAVS) Regulates the Maturation of NK Cells. J Immunol. (2015) 195:2157–67. 10.4049/jimmunol.1402060 26232430 PMC4709023

[5] Liu S Cai X Wu J Cong Q Chen X Li T. Phosphorylation of innate immune adaptor proteins MAVS, STING, and TRIF induces IRF3 activation. Science. (2015) 347:aaa2630. 10.1126/science.aaa2630 25636800

[6] Pomerantz JL Baltimore D. NF-kappaB activation by a signaling complex containing TRAF2, TANK and TBK1, a novel IKK-related kinase. EMBO J. (1999) 18:6694–704. 10.1093/emboj/18.23.6694 10581243 PMC1171732

[7] Ma X Helgason E Phung QT Quan CL Iyer RS Lee MW. Molecular basis of Tank-binding kinase 1 activation by transautophosphorylation. Proc Natl Acad Sci USA. (2012) 109:9378–83. 10.1073/pnas.1121552109 22619329 PMC3386122

[8] Hacker H Karin M. Regulation and function of IKK and IKK-related kinases. Sci STKE. (2006) 2006:re13. 10.1126/stke.3572006re13 17047224

[9] Zhong B Yang Y Li S Wang YY Li Y Diao F. The adaptor protein MITA links virus-sensing receptors to IRF3 transcription factor activation. Immunity. (2008) 29:538–50. 10.1016/j.immuni.2008.09.003 18818105

[10] Jin L Getahun A Knowles HM Mogan J Akerlund LJ Packard TA. STING/MPYS mediates host defense against Listeria monocytogenes infection by regulating Ly6C(hi) monocyte migration. J Immunol. (2013) 190:2835–43. 10.4049/jimmunol.1201788 23378430 PMC3593745

[11] Sun W Li Y Chen L Chen H You F Zhou X. ERIS, an endoplasmic reticulum IFN stimulator, activates innate immune signaling through dimerization. Proc Natl Acad Sci USA. (2009) 106:8653–8. 10.1073/pnas.0900850106 19433799 PMC2689030

[12] Abe T Barber GN. Cytosolic-DNA-mediated, STING-dependent proinflammatory gene induction necessitates canonical NF-kappaB activation through TBK1. J Virol. (2014) 88:5328–41. 10.1128/JVI.00037-14 24600004 PMC4019140

[13] McWhirter SM Fitzgerald KA Rosains J Rowe DC Golenbock DT Maniatis T. IFN-regulatory factor 3-dependent gene expression is defective in Tbk1-deficient mouse embryonic fibroblasts. Proc Natl Acad Sci USA. (2004) 101:233–8. 10.1073/pnas.2237236100 14679297 PMC314168

[14] Perry AK Chow EK Goodnough JB Yeh WC Cheng G. Differential requirement for TANK-binding kinase-1 in type I interferon responses to toll-like receptor activation and viral infection. J Exp Med. (2004) 199:1651–8. 10.1084/jem.20040528 15210743 PMC2212814

[15] Matsui K Kumagai Y Kato H Sato S Kawagoe T Uematsu S. Cutting edge: role of TANK-binding kinase 1 and inducible IkappaB kinase in IFN responses against viruses in innate immune cells. J Immunol. (2006) 177:5785–9. 10.4049/jimmunol.177.9.5785 17056502

[16] Yan C Xiao J Li J Chen H Liu J Wang C. TBK1 of black carp plays an important role in host innate immune response against SVCV and GCRV. Fish Shellfish Immunol. (2017) 69:108–18. 10.1016/j.fsi.2017.08.016 28821402

[17] Zhang DL Yu DH Chen J Fan S Wang ZY. Expression profiles and interaction suggest TBK1 can be regulated by Nrdp1 in response to immune stimulation in large yellow croaker Larimichthys crocea. Fish Shellfish Immunol. (2015) 46:745–52. 10.1016/j.fsi.2015.08.013 26291490

[18] Feng X Su J Yang C Yan N Rao Y Chen X. Molecular characterizations of grass carp (Ctenopharyngodon idella) TBK1 gene and its roles in regulating IFN-I pathway. Dev Comp Immunol. (2014) 45:278–90. 10.1016/j.dci.2014.03.018 24704212

[19] Li S Lu LF LaPatra SE Chen DD Zhang YA. Zebrafish STAT6 negatively regulates IFNphi1 production by attenuating the kinase activity of TANK-binding kinase 1. Dev Comp Immunol. (2017) 67:189–201. 10.1016/j.dci.2016.10.003 27743998

[20] Sun F Zhang YB Liu TK Gan L Yu FF Liu Y. Characterization of fish IRF3 as an IFN-inducible protein reveals evolving regulation of IFN response in vertebrates. J Immunol. (2010) 185:7573–82. 10.4049/jimmunol.1002401 21084665

[21] Li J Zhang H Lin D Wu J Wang C Xie X. Spatiotemporal distribution and assemblages of fishes below the lowermost dam in protected reach in the Yangtze River main stream: implications for river management. Biomed Res Int. (2016) 2016:4290793. 10.1155/2016/4290793 27843943 PMC5098102

[22] Li X Li YR Chu L Zhu R Wang LZ Yan YZ. Influences of local habitat, tributary position, and dam characteristics on fish assemblages within impoundments of low-head dams in the tributaries of the Qingyi River, China. Dongwuxue Yanjiu. (2016) 37:67–74. 10.13918/j.issn.2095-8137.2016.2.67 27029863 PMC4848416

[23] Winton J Batts W deKinkelin P LeBerre M Bremont M Fijan N. Current lineages of the epithelioma papulosum cyprini (EPC) cell line are contaminated with fathead minnow, Pimephales promelas, cells. J Fish Dis. (2010) 33:701–4. 10.1111/j.1365-2761.2010.01165.x 20497291

[24] Lu LF Li S Lu XB LaPatra SE Zhang N Zhang XJ. Spring viremia of carp virus N protein suppresses fish IFNphi1 production by targeting the mitochondrial antiviral signaling protein. J Immunol. (2016) 196:3744–53. 10.4049/jimmunol.1502038 26994222

[25] Lu LF Li S Wang ZX Du SQ Chen DD Nie P. Grass carp reovirus VP41 targets fish MITA to abrogate the interferon response. J Virol. (2017) 91:e00390–17. 10.1128/JVI.00390-17 28446676 PMC5487562

[26] Biacchesi S LeBerre M Lamoureux A Louise Y Lauret E Boudinot P. Mitochondrial antiviral signaling protein plays a major role in induction of the fish innate immune response against RNA and DNA viruses. J Virol. (2009) 83:7815–27. 10.1128/Jvi.00404-09 19474100 PMC2715792

[27] Takahashi A Kobayashi Y Mizusawa K. The pituitary-interrenal axis of fish: a review focusing on the lamprey and flounder. Gen Comp Endocrinol. (2013) 188:54–9. 10.1016/j.ygcen.2013.03.005 23524003

[28] Nardocci G Navarro C Cortes PP Imarai M Montoya M Valenzuela B. Neuroendocrine mechanisms for immune system regulation during stress in fish. Fish Shellfish Immunol. (2014) 40:531–8. 10.1016/j.fsi.2014.08.001 25123831

[29] Gurtler C Bowie AG. Innate immune detection of microbial nucleic acids. Trends Microbiol. (2013) 21:413–20. 10.1016/j.tim.2013.04.004 23726320 PMC3735846

[30] Zevini A Olagnier D Hiscottt J. Crosstalk between Cytoplasmic RIG-I and STING Sensing Pathways. Trends Immunol. (2017) 38:194–205. 10.1016/j.it.2016.12.004 28073693 PMC5329138

[31] Fang R Jiang QF Zhou X Wang CG Guan YK Tao JL MAVS activates TBK1 and IKK epsilon through TRAFs in NEMO dependent and independent manner. Plos Pathog. (2017) 13. 10.1371/journal.ppat.1006720 PMC5699845 29125880

[32] Zhang J Zhang YB Wu M Wang B Chen C Gui JF. Fish MAVS is involved in RLR pathway-mediated IFN response. Fish Shellfish Immunol. (2014) 41:222–30. 10.1016/j.fsi.2014.09.002 25219369

[33] Fitzgerald KA McWhirter SM Faia KL Rowe DC Latz E Golenbock DT IKK epsilon and TBK1 are essential components of the IRF3 signaling pathway. Nat Immunol. (2003) 4:491–6. 10.1038/ni921 12692549

[34] Li S Wang L Berman M Kong YY Dorf ME. Mapping a dynamic innate immunity protein interaction network regulating type I interferon production. Immunity. (2011) 35:426–30. 10.1016/j.immuni.2011.06.014 21903422 PMC3253658

